# Invasion front dynamics in disordered environments

**DOI:** 10.1038/s41598-020-75366-1

**Published:** 2020-10-26

**Authors:** Youness Azimzade, Mahdi Sasar, Iraj Maleki

**Affiliations:** Department of Physics, University of Tehran, 14395-547 Tehran, Iran

**Keywords:** Biophysics, Ecology, Evolution, Physics, Biological physics, Statistical physics, thermodynamics and nonlinear dynamics

## Abstract

Invasion occurs in environments that are normally spatially disordered, however, the effect of such a randomness on the dynamics of the invasion front has remained less understood. Here, we study Fisher’s equation in disordered environments both analytically and numerically. Using the Effective Medium Approximation, we show that disorder slows down invasion velocity and for ensemble average of invasion velocity in disordered environment we have $\bar{v}=v_0 (1-|\xi |^2/6)$ where $|\xi |$ is the amplitude of disorder and $v_0$ is the invasion velocity in the corresponding homogeneous environment given by $v_0=2\sqrt{RD_0}$. Additionally, disorder imposes fluctuations on the invasion front. Using a perturbative approach, we show that these fluctuations are Brownian with a diffusion constant of: $D_{C}= \dfrac{1}{8} \xi ^2\sqrt{RD_0 (1-|\xi |^2/3)}$. These findings were approved by numerical analysis. Alongside this continuum model, we use the Stepping Stone Model to check how our findings change when we move from the continuum approach to a discrete approach. Our analysis suggests that individual-based models exhibit inherent fluctuations and the effect of environmental disorder becomes apparent for large disorder intensity and/or high carrying capacities.

## Introduction

Invasion plays a central role in different biological contexts from the introduction of a population in a new habitat to tumor growth^1,2^. A population capable of consecutive duplication and dispersion can develop a successful invasion where it may pose a threat to existing populations. Invasion separates space into two areas: occupied and unoccupied. The study of invasion inevitably leads to an analysis of the interface between these two areas and how it evolves^3–7^. The speed of this interface is of central importance because it provides an understanding of invasion velocity and mechanisms behind invasion^8,9^.

The spread of populations is a phenomenon that exhibits resemblance with processes that are governed by the reaction-diffusion equations^10^. Invasion of various populations^11^ and tumor growth^6,7,12,13^ is described by different versions of Fisher-Kolomogorov-Petrovsky-Piskunov (FKPP) equation which in its classical form is described as:

1 $$\begin{aligned} \frac{\partial C}{\partial t}=RC(1-C)+\frac{\partial }{\partial x} \left(D\frac{\partial }{\partial x} C\right) \end{aligned}$$

in which *C*(*x*, *t*) is the density of the population, *R* is the growth rate, and *D* is the diffusion constant for the population. Equation (1) represents a diffusion equation with a nonlinear reaction, that leads to the propagation of the Fisher waves. These are traveling-waves as $C(x,t)=C(z)$ with $z=x-vt$ and $C(z)\sim ze^{-\alpha z}$ where $v=2\sqrt{DR}$ is the invasion velocity^14^. While Eq. (1) describes invasion as a deterministic process, populations are composed of discrete individuals exhibiting fluctuations in different aspects. Such fluctuations contribute to invasion dynamics and may even give rise to new phenomena^15^. Birth and death processes are inherently stochastic. To incorporate such fluctuations into deterministic Fisher’s equation, a noise in growth term was introduced^16,17^ as:

2 $$\begin{aligned} \frac{\partial C}{\partial t}=RC(1-C)+\frac{\partial }{\partial x} \left(D\frac{\partial }{\partial x}C\right)+\sqrt{\gamma C(1-C)/K}\eta \end{aligned}$$

where $\gamma >0$ is the strength of the noise, *K* is the *Carrying Capacity* for corresponding environment and $\eta$ is white noise. Adding this noise term leads to emergence of fluctuations in the density profile of invasion front. Among properties of invasion front, *Front Position* which is defined by $C_F=\int _{0}^{\infty }C(x,t)dx$ follows a Langevin equation. Diffusion constant is defined to be $\langle (C_{F} -\bar{C_{F}})^{2} \rangle = 2D_{C}t$ where $\bar{C_{F}}$ stands for ensemble average.

Discrete models have been developed to study invasion as well^18–21^. Stepping Stone Model (SSM) is one of the best-known models to describe population dynamics^22^. SSM governs an integer number of species living in a discrete environment and capable of duplication within and migration between units (demes), which leads to their propagation into available demes^18,22^. This process eventually leads to invasion in patterns similar to FKKP. However, due to their stochastic nature and interactions between individuals, the invasion dynamics can be different^9^. We use this model to check how discrete individuals invade in disordered environments and whether or not results for Fisher’s equation can be generalized to study them.

Fisher postulated that the diffusivity can be spatially disordered^23^, however, few studies have been carried out to address the effect of such disorder on invasion^7,24–29^. Biological entities can invade through environments that are not uniform^30^. Spatial variations in the physical properties of the environment can be considered as a disorder for invading populations^31,32^. Models to understand the effect of irregularity on invasion were introduced as early as 1986^33^. Later studies explored different aspects of invasion in disordered environments and how it affects invasion of different populations both theoretically and experimentally^24–27^. However, to the best of our knowledge, there are two aspects that have not yet been addressed: (i) The effect of disorder on invasion velocity (ii) The dynamics of fluctuations induced by disorder.

Disorder plays a fundamental role in regulating a variety of phenomena in physical systems of fundamental scientific importance, as well as those that are encountered in practice^34,35^. Prominent examples include the influence of disorder on flow, transport, reaction, and deformation properties of materials, such as porous media^36^ and composite solids^34,35^. More interestingly, propagation of waves, and the structure of wavefronts in different systems show sensitivity to disorder^7,37,38^. Effective Medium Approximation (EMA) has been proposed as a means to study propagation phenomena in disordered environments^39^. This approach suggests that a spatially disordered environment can be replaced with a hypothetical homogeneous one with unknown constants and the deterministic Fisher’s equation can still describe the dynamics of the average ensemble of propagating waves.

In this Letter, we study the effect of spatial disorder of the diffusion coefficient on the invasion of Fisher’s equation and SSM. We calculate the velocity of Fisher’s equations using EMA. Then we obtain the dynamics of fluctuations using a perturbative approximation and perform numerical analysis to check the validity of our findings. Additionally, we use SSM to study how the disorder affects the invasion of discrete individuals.

## Models

### Fisher’s equation

**Figure 1:**
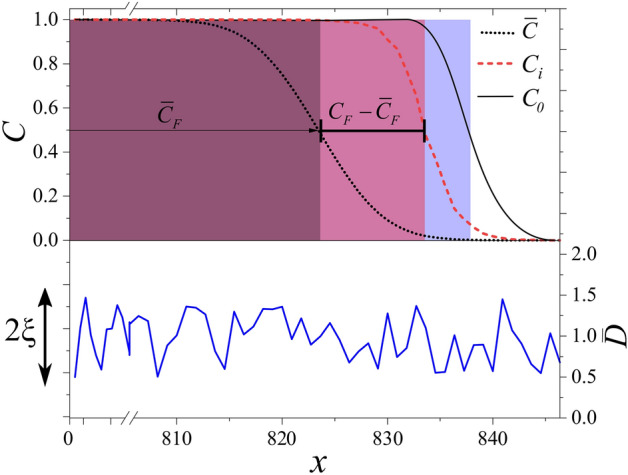
The Fisher wave propagation in an environment with a spatially irregular diffusivity $\bar{D}$ (shown by blue solid line) where intensity of disorder is determined by $\xi$ as $\bar{D}(x)=D_0 (1+\xi f(x))$ with *f* being white noise with uniform distribution in the range of [− 1, 1]. Any single realization of waves in disordered environment ($C_i$) would move slower than unperturbed wave front ($C_0$) that propagates in a uniform environment. One can assign an ensemble average to propagating waves and Effective Medium Approximation (EMA) suggests that average wave fronts, $\bar{C}$, follows a Fisher’s equation with effective diffusion constant, $D_e$.

Changing the migration probability in individual scale leads to variation in the diffusion coefficient in the differential equation that describes the density at mesoscopic scales^40,41^. As such, we translate randomness in the jumping probability to those in the diffusion coefficient in the FKPP equation. Thus, the dynamics of the invasion process is described by

3 $$\begin{aligned} \frac{\partial C}{\partial t}=RC(1-C)+\frac{\partial }{\partial x}\big ( D_0[1+\xi f(x)] \frac{\partial }{\partial x} C\big ) \end{aligned}$$

where a fluctuating diffusion constant as $\bar{D}(x)=D_0[1+\xi f(x)]$ substitutes previously uniform diffusion constant at Eq. (1), and *f* is a uniform white noise in the range $[-1,1]$. $\xi$ represents the amplitude of disorder in *D* and as noise that satisfies $\int \xi (x) \xi (x')dx=\delta (x-x')$ where $\delta (x-x')$ stands for the Dirac delta function and the noise term has the dimension of $m^{1/2}$ where *m* stands for meter as length unit. We study the motion of the invasion front using the front position, defined by, $C_F=\int _0^\infty C(x,t)dx$, which is shown in Fig. 1.

### Stepping stone model

**Figure 2:**
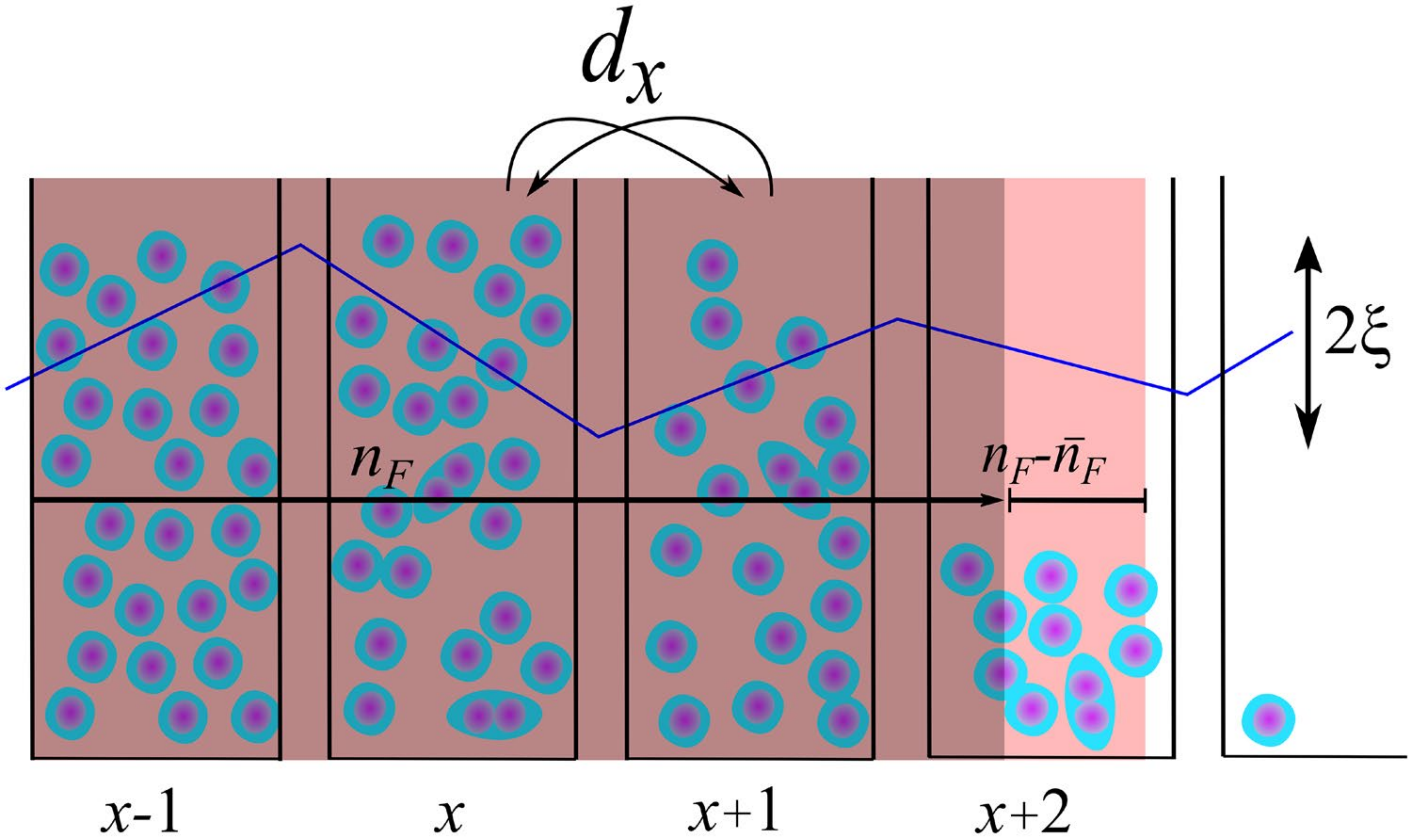
Schematic illustration of the Stepping Stone Model (SSM) with a spatially irregular migration rate, $d_x$ (blue solid line). We show the normalized number of species in deme *x* at time *t* by $n(x,t)=N(x,t)/K$ where *N*(*x*, *t*) is the number species at (*x*, *t*) and *K* is the carrying capacity. We define front position as $n_F(t)=\Sigma _{x=0}^\infty n(x,t)$ and invasion velocity as $v=\frac{d n_F(t)}{d t}$. The averaged invasion velocity is defined based on ensemble average of front position as $\bar{v} =\frac{d \bar{n}_F(t)}{d t}$. We also analyze front position fluctuations, $n_F(t)-\bar{n}_F(t)$.

In the traditional version of a one-dimensional (1D) SSM without mutation^22^, the entities are living within units (demes) of a 1D lattice, where each unit has the carrying capacity of *K*, and is able to duplicate with a probability $r=r_0[1-n(x,t)]$, $n(x,t)=N(x,t)/K$ is the normalized number of entities, and *N*(*x*, *t*) is the number of entities in unit *x* at time *t*. Entities can jump (migrate) to their nearest neighbors with a probability $d_0$^18^. To study the effect of the heterogeneity, we consider a spatially irregular probability for jumping from *x* to $(x+1)$, and vise Versa, given by, $d_x=r_0[1+\xi f(\mathbf{x})]$; see Fig. 2. To induce time passage we select each individual randomly and it will duplicate with the probability of *r*. Then, independent of duplication, it will migrate with the probability of $d_x$. Respectively, the both duplication and migration processes follow a Binomial distribution. For considerably small values of *r* and $d_x$, the Binomial distribution converges to Poisson distribution^42^. To analyze wanderings of the invasion front, we study $n_F(t)-\bar{n}_F(t)$, where the *front position* is defined as, $n_F(t)=\Sigma _{x=0}^\infty n(x,t)$, where $\bar{n}_F(t)$ is the average over the ensemble at the time *t*.

## Results

### Invasion velocity

In what follows, we will analyze the effect of disorder on invasion velocity using the aforementioned continuum and discrete approaches.

#### Effective medium approximation

The main idea behind the EMA is that disordered environments can be described by effective properties which can be obtained through a self-consistent approach^39^. Then, the ensemble average of propagating waves can be described by such effective properties. Since invasion velocity depends on the diffusion constant, to find the invasion velocity, we need to calculate the effective diffusion constant. Using EMA we find that the effective diffusion constant is $D_{e}=D_0(1-|\xi |^2/3)$ (see Material and Methods). EMA suggests that the ensemble average of invasion waves still can be described by the Fisher’s equation. In other words, for the averaged invasion velocity we should have $\bar{v}=2\sqrt{RD_{e}}=2\sqrt{RD_0(1-|\xi |^2/3)}$. Assuming $\xi<<1$, using a standard Taylor expansion as $\sqrt{1-u^2}=1-u/2$, one has $\bar{v}=v_0 (1-|\xi |^2/6)$ with $v_0=2\sqrt{RD_0}$.

#### Numerical analysis of Fisher’s equation

**Figure 3:**
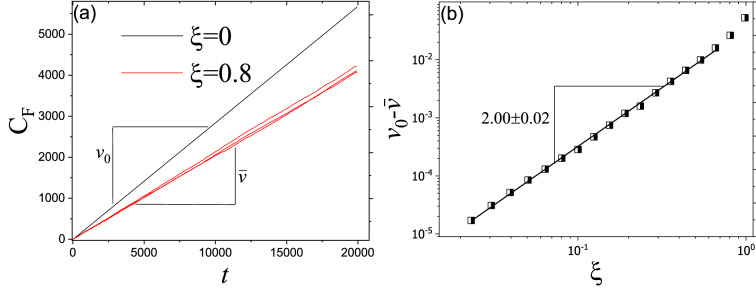
(**a**) $C_F$ versus time for different values of $\xi$. As we increase the amplitude of disorder, $\xi$, $C_F$ exhibit fluctuations. But still it is possible to define an average invasion velocity, $\bar{v}$. (**b**) Decrease in averaged invasion velocity versus $\xi$. As the EMA suggests, invasion velocity decreases as $v_0-\bar{v} \propto \xi ^{2\pm 0.02}$.

As shown in Fig. 1 and suggested by EMA, disorder slows down the propagation of Fisher waves. Despite exhibiting fluctuations, still one can assign an average invasion velocity to Fisher waves in disordered environments (see Fig. 3a). To numerically quantify the effect of disorder, we calculate the averaged invasion velocity of Fisher waves, $\bar{v}$, for different values of $\xi$. Fig. 3b shows that for changes in invasion velocity we have: $v_0 -\bar{v} \propto \xi ^{2.00\pm 0.02}$. This result is in agreement with what EMA suggests.

#### Stepping stone model

**Figure 4:**
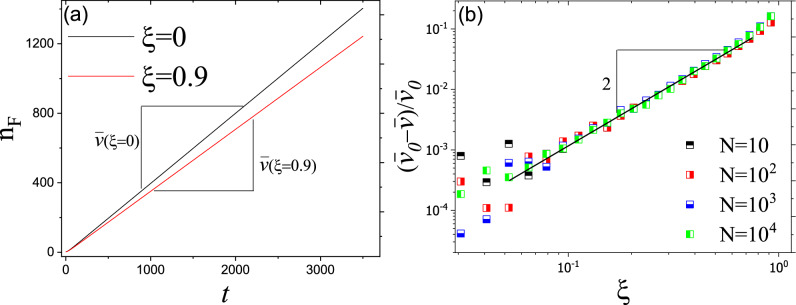
(**a**) $n_F$ versus time for different values of $\xi$. As we increase the amplitude of disorder, $\xi$, $n_F$ increases slower. (**b**) Decrease in normalized invasion velocity versus $\xi$. Similar to Fisher’s equation analysis, invasion velocity decreases as $(\bar{v} _0-\bar{v})/\bar{v}_0 \propto \xi ^2$.

In this part, we study how the disorder affects invasion velocity in an individual-based model. A comparison between the front position of different environments suggests that disorder reduces invasion velocity in SSM (see Fig. 4a). Since the individual-based model contains inherent randomness in events of duplication and migration, there is a fluctuation in a homogeneous environment. As we increase the intensity of disorder, gradually, the fluctuations imposed by the environment become larger and dominant. As Fig. 4b shows, for considerably large values of $\xi$, for changes in normalized invasion velocity $(\bar{v} _0-\bar{v})/\bar{v}_0 \propto \xi ^2$ which is in agreement with results for Fisher’s waves (since invasion velocity depends on carrying capacity, we showed normalized invasion velocity in order to have comparable data).

### Dynamics of fluctuations in the front position

As mentioned earlier, disorder imposes fluctuation on the invasion front. In this part, we quantify these fluctuations.

#### Perturbation approach

The exact mathematical analysis of Eq. (3) is undeniably a formidable task as evidenced by the corpus of references on this subject. However, it can be shown that valuable insight into the dynamics of the problem could be obtained using a series of subtle mathematical maneuvers. Our analytical approach relies on two main assumptions that were previously shown to be practical^17^. First, in agreement with the existing literature, we assume a linear version of Eq. (3) by removing the $C^2$ term. While considering a linear version is a rather standard practice in literature, it should be noted that our purpose is to analyze the invasion front where $C<<1$ for which ignoring the non-linear term seems more rational. As the second assumption, we perform a perturbation analysis which is also been used previously^17^. To do so, we choose a comoving reference frame and calculate fluctuations of invasion front in respect to this moving frame (see Material and Methods). Based on our results, disorder imposes a Brownian fluctuation to invasion front with the diffusion constant of: $D_{C}(t\rightarrow \infty ) =\dfrac{1}{8} \xi ^2 \sqrt{R D_e} =\dfrac{1}{8} \xi ^2 \sqrt{R D_0(1- |\xi |^2/3)}$.

#### Numerical analysis of Fisher’s wave

**Figure 5:**
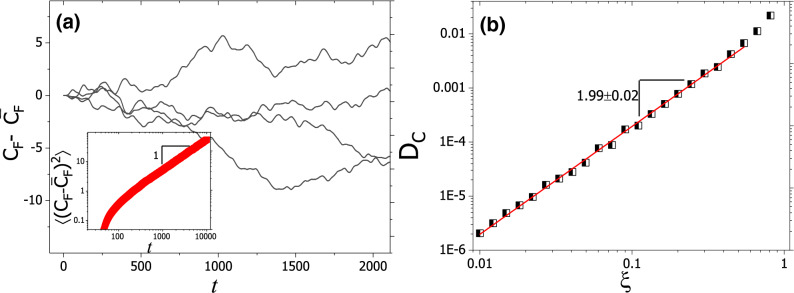
(**a**) The evolution of $\langle (C_F-\bar{C}_F)\rangle$, with the inset showing the evolution of $\langle (C_F-\bar{C}_F)^2\rangle$, indicating Brownian dynamics of the fluctuations of $\langle (C_F-\bar{C}_F)\rangle$. Thus, we can define a diffusion constant, $D_C$, for invasion front fluctuations. (**b**) $D_C$ versus $\xi$ for $R=0.01$. The most accurate yields is, $D_C\propto \xi ^{1.99pm 0.02}$.

In this part, we numerically analyze two aspects of these fluctuations. First, we check if fluctuations are Brownian. Fig. 5a indicates that the environmental disorder leads to fluctuation of $C_F(t)$. The $\log -\log$ diagram of $\langle \big (C_F(t)-\bar{C}_F(t)\big )^2\rangle$ as a function of time in the inset of Fig. 5a confirms a linear behavior with a slope of one. Thus, we can write, $\langle \big ( C_F(t)-\bar{C}_F(t)\big ) ^2\rangle \sim D_C t$, where $D_C$ is the invasion front diffusion coefficient. The first implication of the results is that the fluctuations in the front position are Brownian. As the second aspect, we analyze the dependency of these fluctuations on the intensity of the disorder. Figure 5b indicates that we have: $D_C\sim \xi ^{1.99\pm 0.02}$. As such, predictions of the perturbation holds.

#### Stepping stone model

**Figure 6:**
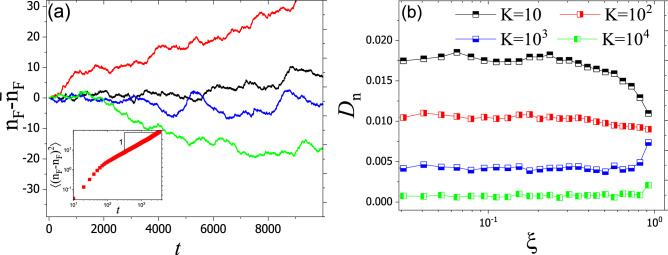
(**a**) Dynamic evolution of of $n-\bar{n}$. The inset shows the evolution of $\langle (n-\bar{n})^2\rangle$ for $K=10$. The slope is equal to one, indicating Brownian dynamics with $\langle (n-\bar{n})^{2}\rangle \sim D_n t$ where $D_n$ is the invasion front diffusion constant. (**b**) $D_n$ versus $\xi$. As we increase the carrying capacity, dependency of front diffusion constant on intensity of disorder changes.

We finally analyze the fluctuations of invasion front of SSM due to disorder. These fluctuations are Brownian (see Fig. 6) and one can define a diffusion constant for them as $\langle \big ( n_F(t)-\bar{n}_F(t)\big ) ^2\rangle \sim D_n t$. However, the invasion front diffusion constant, $D_n$, does not exhibit direct dependence on intensity of disorder. The reason behind this difference relies on inherent fluctuations of SSM due to randomness in migration and duplication.

## Discussion

Quantitative understanding of the invasion is of practical and theoretical importance across different fields. The deterministic version of Fisher’s equation (Eq. 1) has been used widely in the literature, however, stochastic versions describe a more realistic scenario and were introduced later on^43^. The current understanding of stochastic FKPP is shaped by studies that have focused on fluctuating reaction term (Eq. 2). Respectively, the effect of fluctuating diffusion constant has remained relatively unexplored. Particularly, the question of how fast these traveling waves move in presence of disorder has remained unclear.

To address this problem, we studied how the existence of disorder, which seems to be a common feature among different habitats, affects invasion velocity in Fisher’s waves. Previous results suggested that disorder decreases invasion velocity^7^ but the effect has not been quantified. We used both numerical analysis and analytical approximation (EMA) to quantify the effect of disorder on invasion velocity. Our results, while confirming each other, suggest that invasion velocity decreases as $\bar{v}= v_0 (1-|\xi |^2/6)$ for Fisher’s waves. Our finding not only confirms previous results on the effect of environmental disorder on invasion velocity but also quantifies it.

There is widespread and legitimate concern regarding applying model results to realistic systems. Small differences between model assumptions and realistic systems may lead to different behavior in each case. Similarly, a relevant concern regarding Fisher’s equation is that whether or not results obtained by a continuum model can be applied to real systems that are composed of discrete individuals. To address this concern, we studied the effect of disorder on invasion in individual-based models using SSM. Our result suggest that invasion velocity in SSM has a similar dependency on intensity of disorder as $(\bar{v}_0-\bar{v})/\bar{v}_0\propto \xi ^2$ for large values of $\xi$ and/or *K*. This consistency between results of a continuum and discrete models suggests that the obtained effect of disorder on invasion velocity may describe real systems as well. It should be noted that similar to other continuum approaches, Fisher’s equation works well when the carrying capacity is high and processes such as random walks can be described by diffusion. On the other hand, computation cost significantly increases for SSM with large values of *N*. Respectively, it is more appropriate for populations with smaller *N*.

For traveling waves that can be described by Eq. (2), fluctuations of invasion front are inversely regulated by *N*. Thus, as we increase *N* (with the same magnitude for noise strength, $\gamma$), front fluctuations decrease. As a result, noisy reaction term has a relevant effect mainly for smaller values of *N*. On the other hand, for the environmental disorder, the effect of disorder is independent of carrying capacity. As such, disorder plays a relevant role in populations with different carrying capacities and may become the dominant factor for high values of *N* where the fluctuations due to randomness of duplication are small. Respectively, our results emphasize on the importance of environmental disorder and it paves a way for further research.

In the later part, we studied the effect of disorder on front position fluctuations as well. For Fisher’s equation, we have built upon the pioneering work done by Mikhailov et al.^43^ to isolate the contribution of the fluctuating diffusion constant to the position of the front. This allows us to obtain an expression for the fluctuation of the front position and ascertain that the front position performs a Brownian random walk. A similar approach was also undertaken in the study by Birzu et al.^17^. The novelty and major difference between our analysis and that of the aforementioned works is that we do not neglect the explicit time dependence of the solution in our approach and study the effects of the fluctuating diffusion constant as a perturbation on the full (and explicitly time-dependent) solution of the linearized Fisher’s equation.

Our result, while confirming each other, show that disorder leads to Brownian fluctuations of invasion front. These fluctuations can be described by a diffusion constant for which we found: $D_C \propto \xi ^{1.99\pm 0.02}$. Since the evolutionary processes associated with invasion are highly sensitive to the invasion front structure^17^, our results suggest that disorder may change the composition of invading populations as well.

Finally, we studied the effect of disorder on the invasion front of SSM. Due to random selections, SSM exhibits inherent fluctuations that are larger than the effects of the disorder. Respectively, the effect of disorder on $D_n$ remained unclear in the studied parameter space.

## Material and method

### Effective medium approximation

For a linearized version of Fisher , we first obtain the effect of disorder on invasion velocity using Effective Medium Approximation (EMA). Using the EMA approach, we can replace the spatially irregular diffusion constant with a uniform one in which the effective diffusivity is everywhere equal to $D_{e}$ and can replace $D_0(1+\xi f(x))$ in Eq. (2)^44^. To obtain $D_{e}$, one needs to discretize Eq. (2) using a finite-difference method, which leads to the following equation for the population density^39^:

4 $$\begin{aligned} \frac{\partial C_i(t)}{\partial t}=\sum _{j\in \{i\}}W_{ij}[C_j(t)-C_i(t)]+RC_i(t) \;, \end{aligned}$$

where *j* belongs to nearest neighbors of *i* and $W_{ij}=D_{ij}/\delta ^2$ stands for the density flow rate between units *i* and *j* with distance of $\delta$. Due to spatially irregular diffusion constant we have $W_{ij}=W_0(1+\xi f_{ij})$. Following^44^, one has

5 $$\begin{aligned} D_e=\bigg ( \int _{0}^{\infty } \frac{g(w) dw}{w} \bigg )^{-1} \end{aligned}$$

where *g*(*w*) is probability density function for $W_{ij}$. Applying this approach to our case leads to

6 $$\begin{aligned} D_{e}=D_0(1-|\xi |^2/3) \end{aligned}$$

where $|\xi |$ stands for dimensionless magnitude of $\xi$ ($\xi ^2$ has a physical dimension of length, meter).

### Perturbation analysis for invasion front fluctuations

The first step towards the study of dynamics of a propagating front is linearizing (3) by neglecting the $C^2$ term in an environment with effect diffusion constant, $D_e$, as:

7 $$\begin{aligned} \frac{\partial C}{\partial t}=RC+\frac{\partial }{\partial x} \big ((D_e + \xi f(x)) \frac{\partial }{\partial x} C\big ) \end{aligned}$$

This is based on the fact that near the front, the cell density is $C \ll 1$. In other words, focusing on the dynamics of the front position, automatically grants us the possibility of linearizing (3).

The construction of the solution can be proceeded according to a valuable insight given in the classic paper^43^, where the particle density is written as follows

8 $$\begin{aligned} C(\zeta ,t) \approx C_0(\zeta +\eta (t),t)+ \delta C_1(\zeta ,t) \end{aligned}$$

where *C* is written in the comoving frame and $\zeta = x-vt$. It is assumed that $\delta C_1 \ll 1$ and in the same order as the perturbing function. So that terms containing *f*(*x*) and $\delta C_1$ can be neglected. Furthermore, $C_0$ is assumed to satisfy the linearized Eq. (3) with $\xi =0$, i.e.

9 $$\begin{aligned} \frac{\partial C_0(\zeta ,t)}{\partial t}-\hat{\Gamma } C_0(\zeta )=\,& {} \frac{\partial C_0(\zeta ,t)}{\partial t} \nonumber \\&-\bigg (D_e \frac{d^2}{d\zeta ^2} + v\frac{d}{d\zeta } + R\bigg )C_0(\zeta ,t) = 0 \end{aligned}$$

Which has the following solution

10 $$\begin{aligned} C_0(\zeta ,t) = \frac{1}{\sqrt{4\pi D_e t}}e^{-\frac{1}{2}\sqrt{\frac{R}{D_e}}\zeta }e^{-\frac{\zeta ^2}{4D_e t}} \end{aligned}$$

The first term in (8) describes the effects of the perturbing function *f*(*x*) on the position of the propagating front, while the second term shows the change in the shape of the front. This approach has also been employed and well explained in a recent paper^17^. As shown in^17,43^, to determine the effective diffusion coefficient for the fluctuating front, it is sufficient to solve (3) using (8) for $\eta (t)$. Note also that since we are interested in the dynamics of the system in long times $(t \gg \frac{1}{R})$, $v_e$ can be assumed to be equal to $2\sqrt{R D_e}$^45^. Plugging (8) in Eq. (9) expressed in comoving coordinates and considering $\xi =\bar{\xi }/D_e$ yields

11 $$\begin{aligned} \frac{\partial \delta C_1}{\partial t} - \hat{\Gamma }\delta C_1 + \dot{\eta (t)} C_0(\zeta ,t) = \bar{\xi } \bigg (f(\zeta ) C'_0(\zeta ,t)\bigg )' \end{aligned}$$

Noting that the operator $\hat{\Gamma }$ is not self-adjoint (The adjoint of $\hat{\Gamma }$ is: $\hat{\Gamma ^\dagger }=D_e \dfrac{d^2}{d\zeta ^2} - v_e \dfrac{d}{d\zeta } + R$) and following^43^, we multiply Eq. (11) from the left in the eigenfunction of $\hat{\Gamma ^\dagger }$ with 0 eigenvalue (which is $e^{\sqrt{\frac{R}{D_e}}\zeta }$) and integrate. Thus,

12 $$\begin{aligned} {\,} & \int _{-\infty }^{\infty } e^{\sqrt{\frac{R}{D_e}}\zeta }\frac{\partial \delta C_1(\zeta ,t)}{\partial t} d\zeta + \dot{\eta (t)}\int _{-\infty }^{\infty }e^{\sqrt{\frac{R}{D_e}}\zeta }C'_0(\zeta ,t) d\zeta \nonumber \\&\quad = \bar{\xi } \int _{-\infty }^{\infty } e^{\sqrt{\frac{R}{D_e}}\zeta }\bigg (f(\zeta ) C'_0(\zeta ,t)\bigg )'d\zeta \end{aligned}$$

Which yields

13 $$\begin{aligned} \dot{\eta }(t) =\bar{\xi } \dfrac{\int _{-\infty }^{\infty } e^{\sqrt{\frac{R}{D_e}}\zeta }\bigg (f(\zeta ) C'_0(\zeta ,t)\bigg )' d\zeta }{\int _{-\infty }^{\infty }e^{\sqrt{\frac{R}{D_e}}\zeta }C'_0(\zeta ,t) d\zeta } \end{aligned}$$

Which can further be simplified into

14 $$\begin{aligned} \dot{\eta }(t)=\,& {} \bar{\xi }\dfrac{\int _{-\infty }^{\infty } e^{\sqrt{\frac{R}{D_e}}\zeta }f(\zeta ) C'_0(\zeta ,t) d\zeta }{\int _{-\infty }^{\infty }e^{\sqrt{\frac{R}{D_e}}\zeta }C_0(\zeta ,t) d\zeta } \nonumber \\=\,& {} \bar{\xi } e^{-\frac{R t}{4}}\int _{-\infty }^{\infty } e^{\sqrt{\frac{R}{D_e}}\zeta }f(\zeta ) C'_0(\zeta ,t) d\zeta \end{aligned}$$

Or equivalently,

15 $$\begin{aligned} \eta (t) =\bar{\xi } \int _{0}^{t} d\tau e^{-\frac{R\tau }{4}} \int _{-\infty }^{\infty } d\zeta e^{\sqrt{\frac{R}{D_e}}\zeta }f(\zeta ) C'_0(\zeta ,\tau ) \end{aligned}$$

According to^17^, the effective diffusion would be given by

16 $$\begin{aligned} D_{C} = \dfrac{\langle \eta ^2(t) \rangle }{2t} \end{aligned}$$

Or

17 $$\begin{aligned} D_{C}=\,& {} \frac{\bar{\xi }^2}{2t}\int _{0}^{t}d{T_1}\int _{0}^{t}d{T_2}\int _{-\infty }^{\infty } C'_0(\zeta ,T_1)C'_0(\zeta ,T_2) \nonumber \\&\times e^{-\frac{R T_1}{4}}e^{-\frac{R T_2}{4}}e^{2\sqrt{\frac{R}{D_e}}\zeta } d\zeta \end{aligned}$$

where we have performed an ensemble average over $\eta ^2(t)$ using the fact that $\langle f(x)f(y) \rangle = \delta (x-y)$. A numerical calculation of (16) can be readily computed using any mathematical software. However, valuable insight can still be obtained from (17), if we use dimensionless parameters $\tau _i=\frac{T_i}{t}$ and $\sigma =\sqrt{\frac{R}{D_0}}\zeta$. In other words

18 $$\begin{aligned} D_{C}=\,& {} \bar{\xi }^2 \dfrac{\sqrt{R}}{32\pi D^{3/2}_0}\int _{0}^{1}d\tau _1\int _{0}^{1}d\tau _2\int _{-\infty }^{\infty }d\sigma \nonumber \\&\times \dfrac{\left( 1+\dfrac{\sigma }{Rt\tau _1}\right) \left( 1+\dfrac{\sigma }{Rt\tau _2}\right) }{\sqrt{\tau _1\tau _2}}e^{-\frac{\sigma ^2}{4Rt\tau _1}}e^{-\frac{\sigma ^2}{4Rt\tau _2}}e^{\sigma }e^{-R t\frac{(\tau _1+\tau _2)}{4}} \end{aligned}$$

Equation (18) gives the effective diffusion coefficient for the stochastic behavior of the front. For a diffusive behavior, we would expect this effective diffusion coefficient to tend to a constant at large times. At large times, we can approximate the integral as follows,

19 $$\begin{aligned} D_{C} \approx \bar{\xi }^2 \dfrac{\sqrt{R}}{32\pi D^{3/2}_e}\int _{0}^{1}d\tau _1\int _{0}^{1}d\tau _2\int _{-\infty }^{\infty }d\sigma \dfrac{1}{\sqrt{\tau _1\tau _2}}e^{-\frac{\sigma ^2}{4Rt\tau _1}} e^{-\frac{\sigma ^2}{4Rt\tau _2}}e^{\sigma }e^{-R t\frac{(\tau _1+\tau _2)}{4}} \end{aligned}$$

Luckily, Eq. (19) can be evaluated exactly to yield

20 $$\begin{aligned} D_{C}&\approx \bar{\xi }^2 \dfrac{\sqrt{R}}{32\pi D^{3/2}_e} \nonumber \\&\quad \frac{2 \pi (2 R t-1) \text {Erf}\left( \frac{\sqrt{R t}}{2}\right) -2 \pi e^{2 R t} \text {Erf}\left( \frac{3 \sqrt{R t}}{2}\right) +2 \pi e^{2 R t} \text {Erf}\left( \sqrt{2} \sqrt{R t}\right) +8 \sqrt{\pi } e^{-\frac{1}{4} (R t)} \sqrt{R t}-4 \sqrt{2 \pi } \sqrt{R t}}{R t} \end{aligned}$$

Where *Erf*(*x*) is the error function. As $t\rightarrow \infty$ this gives the following simple relation for the diffusion constant for the wave front

21 $$\begin{aligned} D_{C}(t\rightarrow \infty ) = \bar{\xi }^2 \dfrac{\sqrt{R}}{8 D^{3/2}_e} \end{aligned}$$

Substituting $\xi =\bar{\xi }/D_e$ and $D_e=D_0(1-|\xi |^2/3)$, we will get the following beautiful equation for the effective diffusion constant of the front at large times:

22 $$\begin{aligned} D_{C}(t\rightarrow \infty ) =\dfrac{1}{8} \xi ^2 \sqrt{R D_0(1- |\xi |^2/3)}. \end{aligned}$$
